# Hypothyroidism impairs development of the gastrointestinal tract in the ovine fetus

**DOI:** 10.3389/fphys.2023.1124938

**Published:** 2023-03-03

**Authors:** Rhian Young, Dominika Lewandowska, Emily Long, F. B. Peter Wooding, Miles J. De Blasio, Katie L. Davies, Emily J. Camm, Per T. Sangild, Abigail L. Fowden, Alison J. Forhead

**Affiliations:** ^1^ Department of Physiology, Development and Neuroscience, University of Cambridge, Cambridge, United Kingdom; ^2^ Department of Biological and Medical Sciences, Oxford Brookes University, Oxford, United Kingdom; ^3^ Department of Veterinary and Animal Sciences, University of Copenhagen, Copenhagen, Denmark; ^4^ Department of Neonatology, Rigshospitalet, Copenhagen, Denmark; ^5^ Department of Pediatrics, Odense University Hospital, Odense, Denmark

**Keywords:** fetus, gastrointestinal, thyroid hormones, gastrin, growth, maturation, cortisol

## Abstract

Growth and maturation of the fetal gastrointestinal tract near term prepares the offspring for the onset of enteral nutrition at birth. Structural and functional changes are regulated by the prepartum rise in cortisol in the fetal circulation, although the role of the coincident rise in plasma tri-iodothyronine (T3) is unknown. This study examined the effect of hypothyroidism on the structural development of the gastrointestinal tract and the activity of brush-border digestive enzymes in the ovine fetus near term. In intact fetuses studied between 100 and 144 days of gestation (dGA; term ∼145 days), plasma concentrations of T3, cortisol and gastrin; the mucosal thickness in the abomasum, duodenum, jejunum and ileum; and intestinal villus height and crypt depth increased with gestational age. Removal of the fetal thyroid gland at 105–110 dGA suppressed plasma thyroxine (T4) and T3 concentrations to the limit of assay detection in fetuses studied at 130 and 144 dGA, and decreased plasma cortisol and gastrin near term, compared to age-matched intact fetuses. Hypothyroidism was associated with reductions in the relative weights of the stomach compartments and small intestines, the outer perimeter of the intestines, the thickness of the gastric and intestinal mucosa, villus height and width, and crypt depth. The thickness of the mucosal epithelial cell layer and muscularis propria in the small intestines were not affected by gestational age or treatment. Activities of the brush border enzymes varied with gestational age in a manner that depended on the enzyme and region of the small intestines studied. In the ileum, maltase and dipeptidyl peptidase IV (DPPIV) activities were lower, and aminopeptidase N (ApN) were higher, in the hypothyroid compared to intact fetuses near term. These findings highlight the importance of thyroid hormones in the structural and functional development of the gastrointestinal tract near term, and indicate how hypothyroidism *in utero* may impair the transition to enteral nutrition and increase the risk of gastrointestinal disorders in the neonate.

## Introduction

At birth, the supply of nutrients to the offspring switches from a placental to an enteral source. In preparation for this transition, the fetal gastrointestinal tract undergoes a number of structural and functional changes near term (Trahair and Sangild, 1997; Buddington et al., 2012; Frazer and Good, 2022). The mucosa increases in size, vascularity and transporter expression to provide an effective secretory and absorptive surface area; gastrointestinal innervation and motility develops; and gastric acid, intrinsic factor and digestive enzymes are synthetised over the perinatal period. Overall, the offspring develops the capacity for the digestion of milk and absorption of nutrients at birth. Preterm infants commonly have impaired digestive function and are at increased risk of developing necrotising enterocolitis (NEC) and other gastrointestinal abnormalities (Frazer and Good, 2022).

Development of the gastrointestinal tract *in utero* is regulated by hormones and growth factors present in the circulation and in the amniotic fluid swallowed by the fetus (Trahair and Sangild, 1997). In fetal sheep and pigs, the prepartum rise in circulating cortisol promotes growth and maturation of the gastric glands and intestinal villus-crypt structure, and the expression of digestive enzymes in the intestinal brush border (Trahair et al., 1987a, 1987b; Sangild T. et al., 1994, 1995b). Indeed, antenatal treatment with synthetic glucocorticoids, that also bind to the glucocorticoid receptor, reduces the incidence of NEC and improves gastrointestinal outcomes in premature offspring (Jing et al., 2021).

Maturational events induced by cortisol in the fetus near term may be mediated, in part, by other endocrine systems (Fowden et al., 2016). In fetal pigs, cortisol increases the circulating concentration of the gut hormone gastrin (Sangild P. et al., 1994) which may act simply as a marker of gastrointestinal growth and development and/or may contribute to the maturational effects of glucocorticoids on the structure and function of the digestive system. Furthermore, in fetal sheep, cortisol stimulates the production of tri-iodothyronine (T3) from thyroxine (T4) *via* changes in deiodinase enzyme activities in fetal and placental tissues (Forhead et al., 2006). In turn, the prepartum rise in plasma T3 is known to contribute to the maturation of fetal organs, such as the lungs and liver, in preparation for birth (Forhead and Fowden, 2014).

Thyroid hormones are important for intestinal development in rodents around weaning and in amphibians during metamorphosis (Sirakov and Plateroti, 2011; Sirakov et al., 2014; Frau et al., 2017). In rat intestines, increments in type 1 deiodinase (D1), and decrements in type 3 deiodinase (D3), activities occur over the perinatal period to increase the production and decrease the clearance of T3, respectively, within the developing gastrointestinal tract (Bates et al., 1999). Moreover, in a variety of models studied postnatally, rodents deficient in circulating thyroid hormones or their receptors have abnormal intestinal morphology, motility and digestive enzyme expression (Yeh and Moog, 1977; Fraichard et al., 1997; Plateroti et al., 1999, 2001; 2006; Flamant et al., 2002). However, little is known about the role of thyroid hormones in gastrointestinal development before birth in a species that resembles the human fetus more closely in the timing of somatic growth and prepartum maturation.

At birth, lambs are considered to be pre-ruminants. The ruminant stomach consists of four compartments (the rumen, reticulum, omasum and abomasum) where, in the neonate, the abomasum is the largest and equivalent to the stomach of some mono-gastric species, such as humans and carnivores, in structure and function (Hill, 1968). An oesophageal groove delivers milk directly into the abomasum and, therefore, the developing digestive system of the ovine fetus and neonate is similar to that of the human. Ruminal digestion and microbial fermentation, however, are not initiated until the introduction of solid food.

This study investigated the effect of thyroid hormone deficiency on the structure of the gastrointestinal tract and the activity of brush border hydrolases in the ovine fetus in late gestation. It tested the hypothesis that hypothyroidism retards normal growth of the gastrointestinal mucosa and development of digestive enzyme activities near term.

## Materials and methods

### Animals

The study made secondary use of tissue and plasma samples derived from two BBSRC projects investigating the endocrine control of development of fetal lungs and skeletal muscle (Blasio et al., 2015; Davies et al., 2020). All surgical and experimental procedures were carried out in accordance with the United Kingdom Animals (Scientific Procedures) Act 1986 and approval was obtained from the animal ethics committee at the University of Cambridge. A total of 65 pregnant ewes of known gestational age were maintained on 200 g/day concentrates, with hay and water freely available. Food, but not water, was removed from the ewes for 18–24 h before surgery. Each experimental group included a mix of single and twin, and male and female, fetuses.

### Surgical and experimental procedures

Hypothyroidism was induced *in utero* by surgical removal of the fetal thyroid gland using methods described previously (Hopkins and Thorburn, 1972). Under halothane anaesthesia (1.5% halothane in O_2_-N_2_O), fetal thyroidectomy was carried out between 105 and 110 days of gestation (dGA; term ∼145 days). In twin pregnancies, fetuses underwent either thyroidectomy or a sham operation where the thyroid gland was exposed but not removed.

All fetuses were delivered by Caesarean section after barbiturate euthanasia of the ewe and the fetus (200 mg/kg iv sodium pentobarbitone; Pentoject, Animalcare Ltd., York, United Kingdom). Intact fetuses were delivered at four time points in gestation: 100 (n = 12), 115 (n = 5), 130 (n = 23), and 144 (n = 26) dGA. The thyroidectomised (TX) fetuses were delivered at 130 (n = 18) and 144 (n = 22) dGA. At delivery, a blood sample (5 mL) was obtained by venepuncture of the umbilical artery at delivery, and the plasma was stored at −20°C before analysis. The body weight of the fetus was recorded before a variety of organs were collected and weighed, including the whole stomach (all four compartments) and the small intestines.

The abomasum of the stomach was dissected just before the pyloric sphincter; the duodenum was obtained 10 cm from the pyloric sphincter; the ileum was sampled 10 cm from the ileo-caecal junction; and the jejunum was sampled halfway along the total length of the small intestines. Samples of the gastrointestinal tract were both frozen immediately in liquid nitrogen and stored at −80°C before analysis, and fixed in 4% paraformaldehyde (with 0.2% glutaraldehyde in 0.1 M phosphate buffer, pH 7.4) and embedded in paraffin wax. All of the tissues were fixed for no longer than 24 h, stored in 70% ethanol for a similar duration and processed using the same protocol. The samples were orientated in the wax to allow transverse sectioning of the tissue.

### Plasma hormone concentrations

Plasma concentrations of T3 and T4 were measured by RIA (MP Biomedicals, Loughborough, United Kingdom); the minimum level of detection was 0.14 ng/mL for T3 and 7.0 ng/mL for T4. Plasma gastrin was also determined by RIA (Diasource, Oxford Biosystems, Abingdon, United Kingdom) where the lower limit of detection was 5 p.m. Plasma cortisol concentrations were measured using an ELISA kit (MP Biomedicals) where the minimum level of detection was 2.5 ng/mL.

### Analysis of gastrointestinal structure

Sections of the fetal gastrointestinal tract were cut at 5 µm thickness using a rotary microtome. Tissue samples were stained with haematoxylin and eosin, or Alcian Blue, and mounted with coverslips, following standard procedures. All sections were scanned by a digital slide scanner (NanoZoomer, Hamamatsu Photonics, Welwyn Garden City, United Kingdom) and the images were analysed using NDP. view2 software (Hamamatsu Photonics), blind to the experimental group.

**FIGURE 1:**
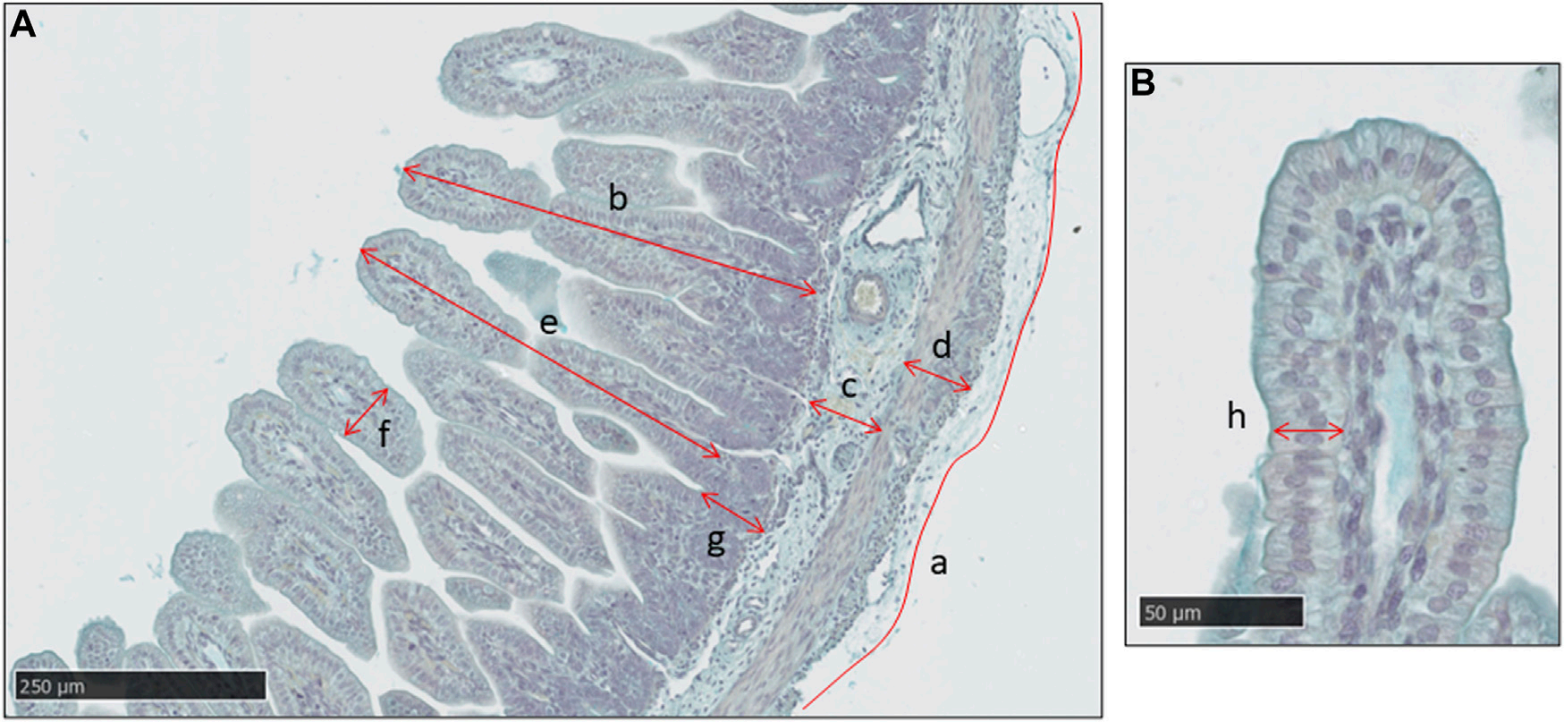
A typical histological image of the jejunum in a sheep fetus at 115 days of gestation, stained with Alcian Blue. **(A)** Measurements made of intestinal outer perimeter (a), thickness of the mucosa (b), sub-mucosa (c) and muscularis propria (d), villus height (e) and width (f) and crypt depth (g). Scale bar = 250 μm. **(B)** Measurement of mucosal epithelial thickness in the villus (h). Scale bar = 50 μm.

Figure 1 shows a typical image of the jejunum with the structural measurements indicated. Mucosal thickness was determined in the abomasum and regions of the small intestines. In the mucosa of the small intestines (duodenum, jejunum, ileum), measurements were made of villus height, villus width, crypt depth and epithelial thickness. The outer intestinal perimeter, and thicknesses of the submucosa and muscularis propria, were also determined. For each variable, apart from the outer perimeter, a mean value was calculated from 5 to 10 measurements distributed across the tissue section. The longest intact villi were measured for height, width and epithelial thickness.

Tissue shrinkage was estimated at 40%–50% using measurements of red blood cell diameter in a random selection of sections across all experimental groups. Although this is a relatively crude measure of tissue shrinkage, no differences were noted between the animal groups. Histological measurements are presented without adjustment for tissue shrinkage.

### Analysis of intestinal enzyme activity

Frozen samples of duodenum, jejunum and ileum were assessed for the activities of enzymes sucrase, maltase, lactase, aminopeptidase A (ApA), aminopeptidase N (ApN) and dipeptidyl peptidase IV (DPPIV) using protocols described previously (Sangild et al., 1995a). The measurements were expressed as U/g tissue.

### Statistical analysis

Data are presented as mean ± SEM. Subsets of the total cohort of fetuses provided data for the measurements of plasma hormones, gastrointestinal weights and histological structure, and brush border enzyme activities. Plasma hormone concentrations below the minimum threshold of detection were assigned the minimum value for data presentation and statistical analysis. The effect of gestational age on variables measured in the intact control fetuses between 100 and 144 dGA was assessed by one-way ANOVA followed by Tukey’s *post hoc* test or one-way ANOVA on Ranks followed by the Dunn’s test, as appropriate (SigmaStat 3.5, Systat Software, San Jose, United States of America). The effect of TX in the fetuses at 130 and 144 dGA was analysed by two-way ANOVA, with treatment and gestational age as factors, followed by Tukey’s *post hoc* test. Significance was considered at *p* < 0.05.

## Results

### Plasma hormone concentrations

**FIGURE 2:**
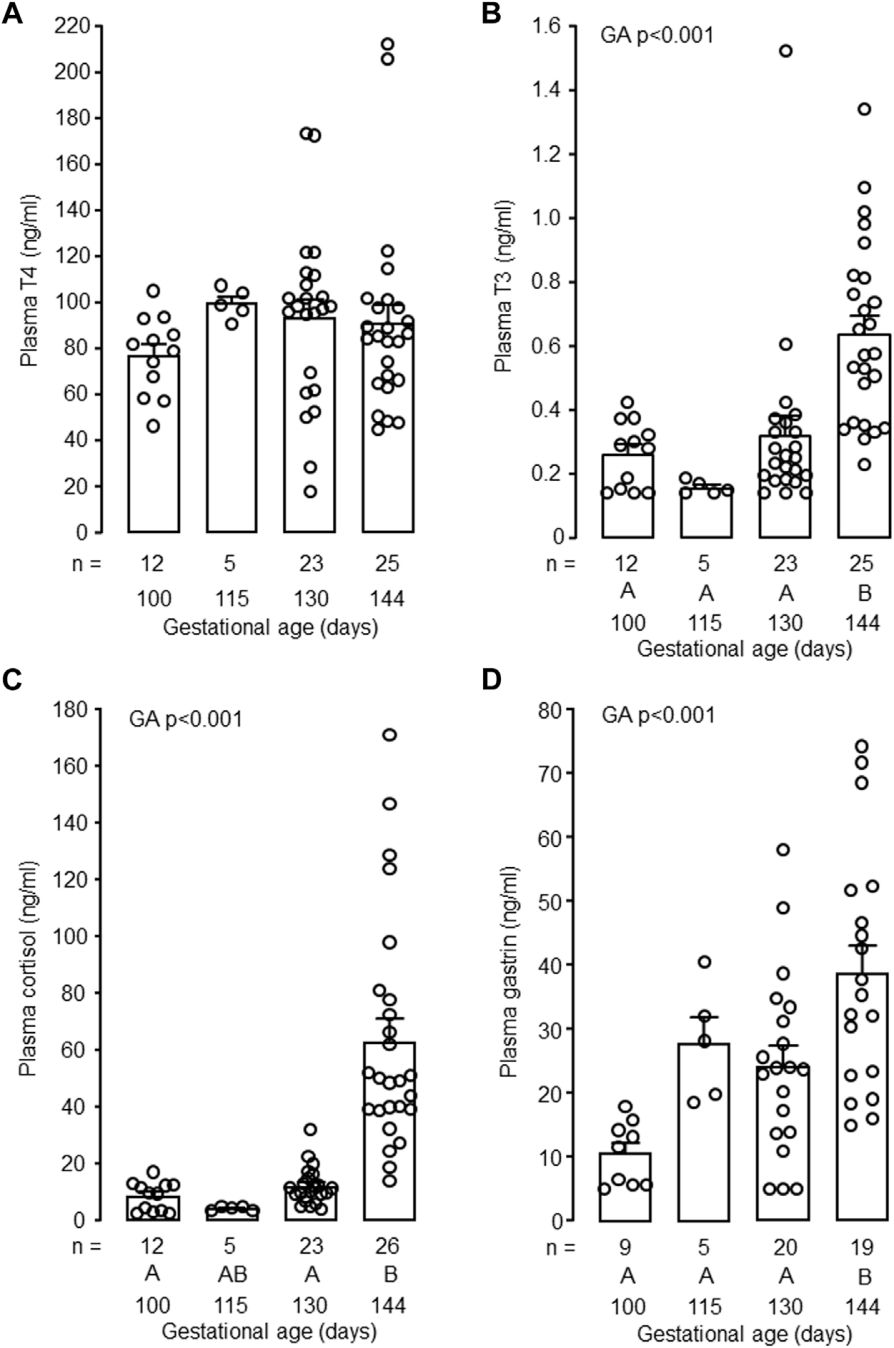
Mean (±SEM) and individual plasma concentrations of **(A)** thyroxine (T4), **(B)** triiodothyronine (T3), **(C)** cortisol and **(D)** gastrin in the intact fetuses studied between 100 and 144 days of gestation. For each parameter measured, values with different letters at the base of the histogram are significantly different from each other (*p* < 0.05, one-way ANOVA). Numbers of fetuses in each group are indicated at the base of the histogram. GA, gestational age.

**FIGURE 3:**
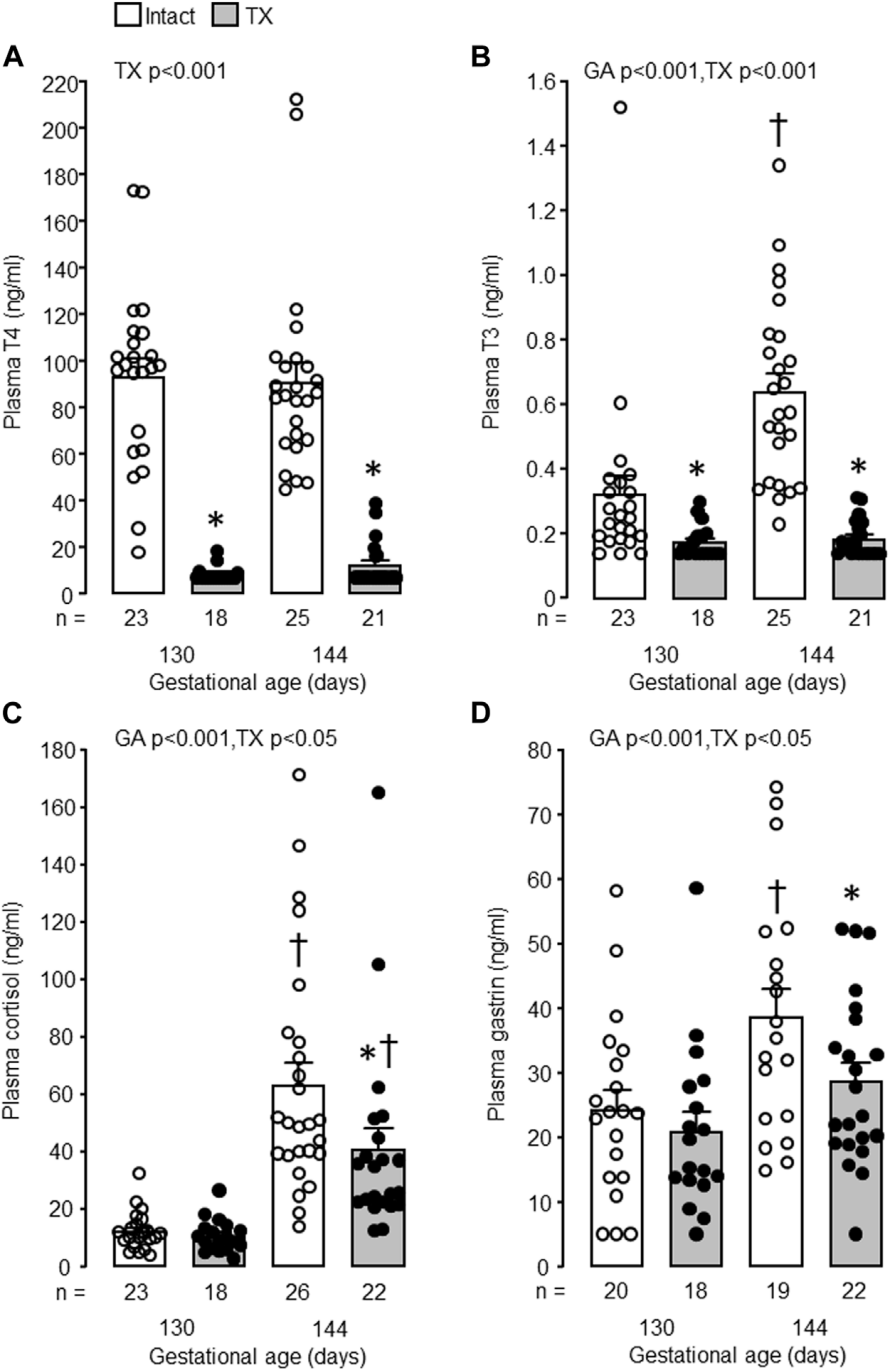
Mean (±SEM) and individual plasma concentrations of **(A)** thyroxine (T4), **(B)** triiodothyronine (T3), **(C)** cortisol and **(D)** gastrin in the intact and TX fetuses at 130 and 144 days of gestation. *, significant difference from intact fetuses at the same gestational age (*p* < 0.05, Tukey’s *post hoc* test following two-way ANOVA); ^†^, significant difference from fetuses of the same treatment at 130 days of gestation (*p* < 0.05, Tukey’s *post hoc* test following two-way ANOVA). Numbers of fetuses in each group are indicated at the base of the histogram. GA, gestational age; TX, thyroidectomy.

In the intact control fetuses, plasma concentrations of T3, cortisol and gastrin increased with gestational age (*p* < 0.001) and were greater in fetuses at 144 dGA compared with those at 130 dGA (*p* < 0.05 in *post hoc* tests, Figures 2B–D). There was no change in plasma T4 concentration between 100 and 144 dGA (Figure 2A). Removal of the fetal thyroid gland decreased plasma T3 and T4 concentrations near to or below the lower limit of assay detection at both 130 and 144 dGA (*p* < 0.001, Figures 3A, B) and prevented the pre-partum rise in plasma T3 (Figure 3B). An interaction was observed between gestational age and treatment in plasma T3 (*p* < 0.001, Figure 3B). Plasma cortisol and gastrin concentrations were affected by both gestational age (*p* < 0.001) and treatment (*p* < 0.05). Plasma cortisol increased between 130 and 144 dGA in both intact and TX fetuses (*p* < 0.05), although the concentration was lower in TX compared to intact fetuses at 144 dGA (*p* < 0.05 in *post hoc* tests, Figure 3C). Hypothyroidism reduced plasma gastrin at 144 dGA (*p* < 0.05) and prevented the rise in concentration observed normally near term (Figure 3D).

### Structure of the gastrointestinal tract

#### Weights of the whole stomach and small intestines

**FIGURE 4:**
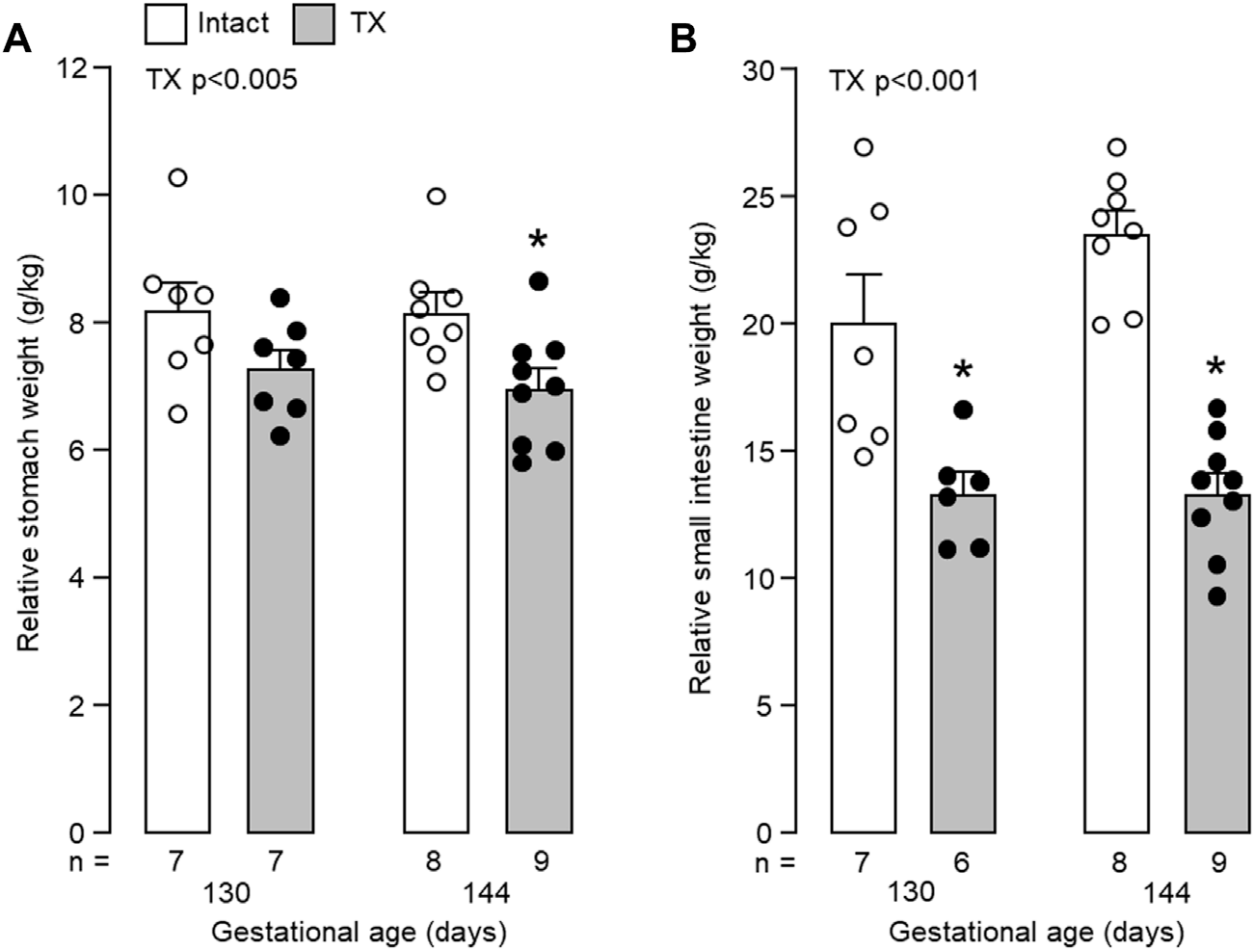
Mean (±SEM) and individual weights of **(A)** the empty stomach and **(B)** the small intestines, expressed relative to body weight, in the fetuses of each experimental group. *, significant difference from intact fetuses at the same gestational age (*p* < 0.05, Tukey’s *post hoc* test following two-way ANOVA). Numbers of fetuses in each group are indicated at the base of the histogram. TX, thyroidectomy.

Fetal body weight increased with gestational age (*p* < 0.05) but was not affected by TX (130 dGA: TX 2.44 ± 0.26 kg versus control 2.41 ± 0.18 kg; 144 dGA: TX 3.08 ± 0.14 kg versus control 3.67 ± 0.25 kg). Growth of the stomach and small intestines between 130 and 144 dGA was impaired by hypothyroidism. Absolute weights of the whole stomach and small intestines were affected by both gestational age (*p* < 0.001) and TX (*p* < 0.005). Absolute stomach and intestine weights were reduced in the TX compared to intact fetuses at 144 dGA (stomach: 21.3 ± 1.0 g versus 29.6 ± 1.7 g; intestines: 41.1 ± 3.1 g versus 86.5 ± 6.9 g, both *p* < 0.05 in *post hoc* tests, n = 8–9), but not at 130 dGA (stomach: 17.4 ± 1.4 g versus 19.8 ± 2.0 g; intestines: 33.3 ± 4.2 g versus 48.6 ± 6.9 g, *p* = 0.07, n = 6–7). The weights of the whole stomach and small intestines, relative to fetal body weight, were decreased by TX (*p* < 0.005)*. Post-hoc* analysis showed that the effect in the stomach was significant at 144 dGA, but not at 130 dGA (*p* < 0.05, Figure 4A). Relative intestinal weights in the TX fetuses were 30%–40% and 40%–50% lower than those in the intact fetuses at 130 dGA and 144 dGA, respectively (*p* < 0.05, Figure 4B).

### Development of the gastrointestinal mucosa

**FIGURE 5:**
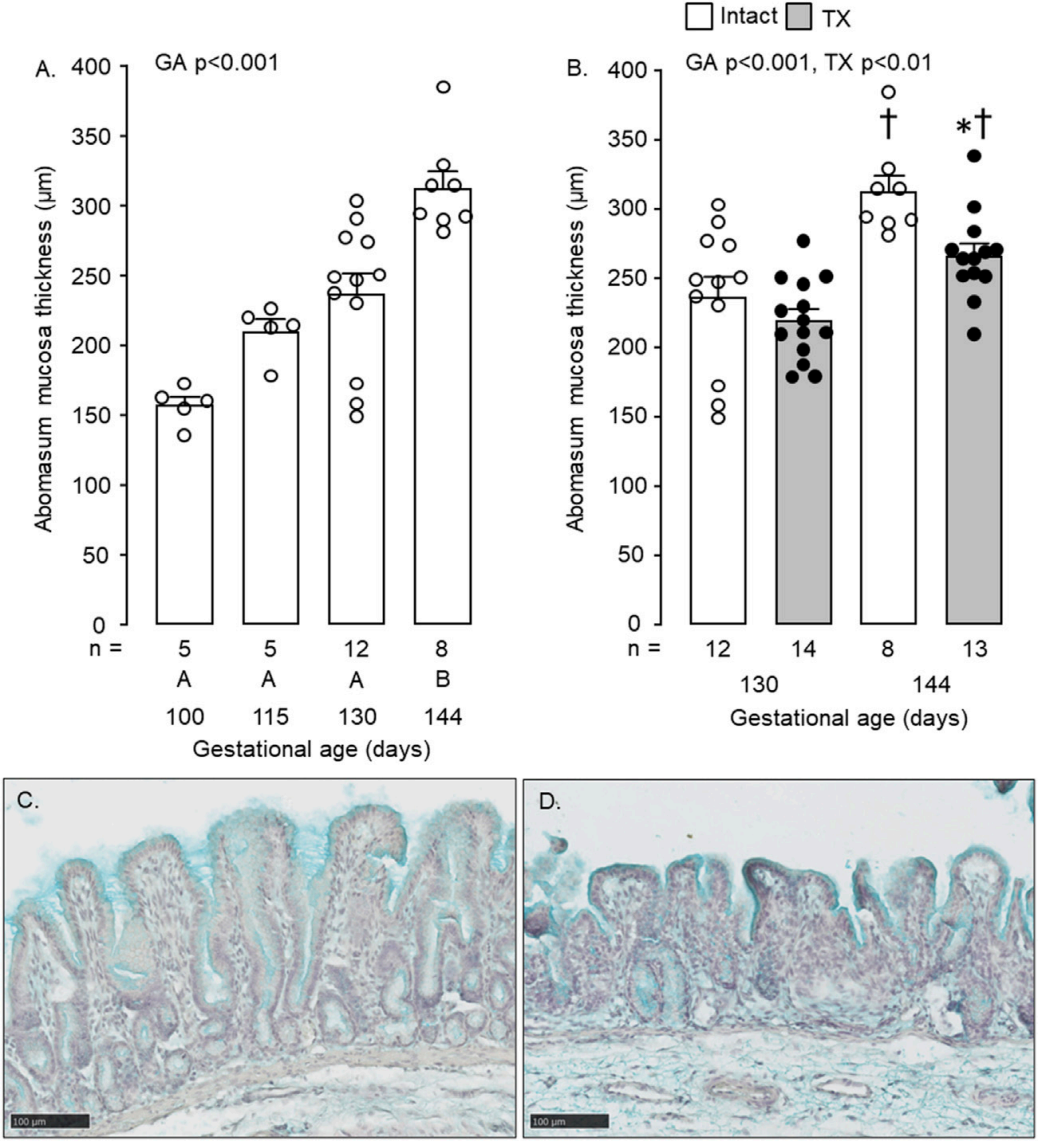
Mean (±SEM) and individual mucosal thickness in the abomasum of **(A)** the intact fetuses studied between 100 and 144 days of gestation and **(B)** the intact and TX fetuses at 130 and 144 days of gestation. In Figure 5A, values with different letters at the base of the histogram are significantly different from each other (*p* < 0.05, one-way ANOVA). In Figure 5B, *, significant difference from intact fetuses at the same gestational age (*p* < 0.05); ^†^, significant difference from fetuses of the same treatment at 130 days of gestation (*p* < 0.05). Numbers of fetuses in each group are indicated at the base of the histogram. GA, gestational age; TX, thyroidectomy. Typical histological images of the abomasum mucosa, stained with Alcian Blue, are shown for intact **(C)** and TX **(D)** twin fetuses at 144 days of gestation. Scale bar = 100 μm.

Mucosal thickness of the abomasum increased with gestational age (*p* < 0.001, Figure 5A). Between 130 and 144 dGA, abomasum mucosal thickness increased in both intact and TX fetuses (*p* < 0.05, Figure 5B), although was lower in the TX compared to intact fetuses at 144 dGA (*p* < 0.05, Figure 5B). Typical images of mucosal development in twin intact and TX fetuses are shown in Figures 5C, D, respectively.

In all regions of the small intestines, increases in outer perimeter, and mucosal thickness, were observed with gestational age in the intact fetuses (*p* < 0.05, Table 1). Hypothyroidism reduced the outer perimeters of the duodenum at 130 dGA, the jejunum at both 130 dGA and 144 dGA, and of the ileum at 144 dGA (*p* < 0.05, Table 1). In the TX compared to intact fetuses, mucosal thickness was lower in the duodenum and jejunum at 130 dGA and in all regions of the small intestines at 144 dGA (*p* < 0.05, Table 1).

**TABLE 1:** Mean (±SEM) measurements of intestinal structure in the fetuses of each experimental group at tissue collection. One-way ANOVA assessed the effect of gestational age in the intact fetuses between 100 and 144 days of gestation: for each parameter measured, values with different superscript letters are significantly different from each other (*p* < 0.05, Tukey’s *post hoc* test). Two-way-ANOVA examined the effects of gestational age and TX in the intact and TX fetuses studied at 130 and 144 days of gestation: *, significant difference from intact fetuses at the same gestational age (*p* < 0.05, Tukey’s *post hoc* test); ^†^, significant difference from fetuses of the same treatment at 130 days of gestation (*p* < 0.05, Tukey’s *post hoc* test). GA, gestational age; TX, thyroidectomy; n, number of animals; NS, not significant.

	Effect of gestational age in intact fetuses					Effect of gestational age and TX						
Gestational age (days)	100	115	130	144	GA	130		144		GA	TX	Interaction
Treatment	intact	intact	intact	intact		intact	TX	intact	TX			
**Duodenum**	n = 5, 6	n = 4	n = 4	n = 4		n = 4	n = 6	n = 4	n = 4			
Outer perimeter (mm)	5.7 ± 0.4^A^	6.4 ± 0.2^A^	9.5 ± 0.2^B^	10.1 ± 0.6^B^	*p* < 0.001	9.5 ± 0.2	7.3 ± 0.5*	10.1 ± 0.6	9.0 ± 1.1	NS	*p* < 0.05	NS
Mucosal thickness (µm)	261.8 ± 24.8^A^	300.0 ± 16.0^A^	385.8 ± 52.9^AB^	458.7 ± 42.3^B^	*p* < 0.005	385.8 ± 52.9	255.0 ± 27.0*	458.7 ± 42.3	291.9 ± 26.6*	NS	*p* < 0.001	NS
Epithelial thickness (µm)	16.5 ± 0.8	16.5 ± 0.7	18.9 ± 0.8	17.3 ± 1.2	NS	18.9 ± 0.8	16.2 ± 0.8	17.3 ± 1.2	17.1 ± 0.8	NS	NS	NS
Submucosal thickness (µm)	174.7 ± 24.6	177.6 ± 21.4	231.3 ± 25.5	232.8 ± 44.7	NS	231.3 ± 25.5	284.6 ± 39.3	232.8 ± 44.7	238.8 ± 44.3	NS	NS	NS
Muscularis propria thickness (µm)	103.7 ± 10.2	107.6 ± 10.0	141.9 ± 19.0	120.4 ± 9.3	NS	141.9 ± 19.0	139.7 ± 22.0	120.4 ± 9.3	125.9 ± 12.3	NS	NS	NS
**Jejunum**	n = 4	n = 4	n = 8, 9	n = 7–9		n = 8, 9	n = 5–7	n = 7–9	n = 4–9			
Outer perimeter (mm)	5.2 ± 0.5^A^	6.8 ± 0.4^AB^	9.1 ± 0.4^BC^	10.1 ± 0.7^C^	*p* < 0.001	9.1 ± 0.4	7.5 ± 0.2*	10.1 ± 0.7	8.2 ± 0.5*	NS	*p* < 0.005	NS
Mucosal thickness (µm)	377.9 ± 22.0^A^	446.4 ± 17.2^A^	652.5 ± 27.2^AB^	842.4 ± 57.5^B^	*p* < 0.001	652.5 ± 27.2	509.1 ± 64.8*	842.4 ± 57.5^†^	671.5 ± 41.2*	*p* < 0.005	*p* < 0.005	NS
Epithelial thickness (µm)	18.0 ± 0.6	17.9 ± 0.3	20.0 ± 1.2	19.2 ± 0.4	NS	20.0 ± 1.2	19.2 ± 1.0	19.2 ± 0.4	19.7 ± 1.0	NS	NS	NS
Submucosal thickness (µm)	64.4 ± 3.9	58.4 ± 2.7	64.4 ± 6.4	58.9 ± 4.2	NS	64.4 ± 6.4	59.5 ± 3.2	58.9 ± 4.2	80.2 ± 5.1*^†^	NS	NS	*p* < 0.05
Muscularis propria thickness (µm)	61.6 ± 5.6	58.9 ± 0.9	75.8 ± 5.6	71.6 ± 7.2	NS	75.8 ± 5.6	64.6 ± 9.1	71.6 ± 7.2	81.2 ± 7.8	NS	NS	NS
**Ileum**	n = 4–10	n = 4, 5	n = 4–8	n = 4–9		n = 4–8	n = 4, 5	n = 4–9	n = 4–9			
Outer perimeter (mm)	6.3 ± 0.3^A^	6.5 ± 0.5^AB^	9.5 ± 0.7^BC^	11.5 ± 1.0^C^	*p* < 0.001	9.5 ± 0.7	7.4 ± 0.3	11.5 ± 1.0	9.3 ± 0.7*	*p* < 0.05	*p* < 0.05	NS
Mucosal thickness (µm)	490.3 ± 33.3^A^	480.2 ± 81.1^A^	713.5 ± 34.0^AB^	915.4 ± 96.6^B^	*p* < 0.001	713.5 ± 34.0	564.7 ± 25.6	915.4 ± 96.6^†^	657.7 ± 64.8*	*p* < 0.05	*p* < 0.005	NS
Epithelial thickness (µm)	22.0 ± 1.1	18.4 ± 0.9	20.9 ± 0.9	19.2 ± 0.6	NS	20.9 ± 0.9	18.9 ± 1.3	19.2 ± 0.6	18.3 ± 1.0	NS	NS	NS
Submucosal thickness (µm)	72.2 ± 6.3^AB^	62.2 ± 6.7^A^	140.0 ± 16.4^AB^	251.5 ± 30.3^B^	*p* < 0.01	140.0 ± 16.4	109.0 ± 27.4	251.5 ± 30.3^†^	138.4 ± 34.4*	*p* < 0.05	*p* < 0.05	NS
Muscularis propria thickness (µm)	87.8 ± 8.1	72.3 ± 6.6	96.1 ± 8.8	89.3 ± 12.8	NS	96.1 ± 8.8	78.9 ± 6.5	89.3 ± 12.8	83.6 ± 9.5	NS	NS	NS

**FIGURE 6:**
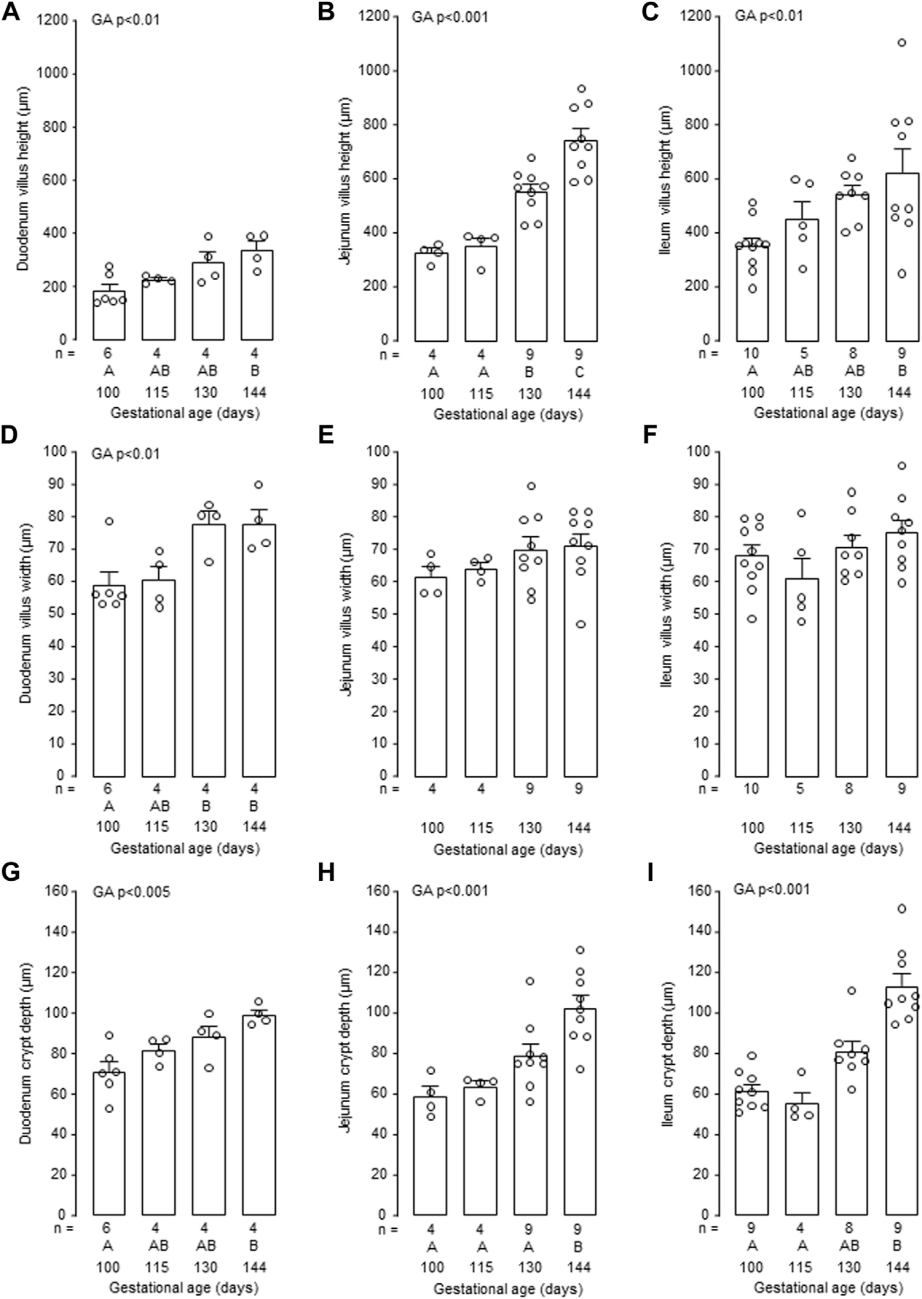
Mean (±SEM) and individual measurements of villus height **(A–C)**, villus width **(D–F)** and crypt depth **(G–I)** in the duodenum, jejunum and ileum of the intact fetuses studied between 100 and 144 days of gestation. For each parameter measured, values with different letters at the base of the histogram are significantly different from each other (*p* < 0.05, one-way ANOVA). Numbers of fetuses in each group are indicated at the base of the histogram. GA, gestational age; TX, thyroidectomy.

**FIGURE 7:**
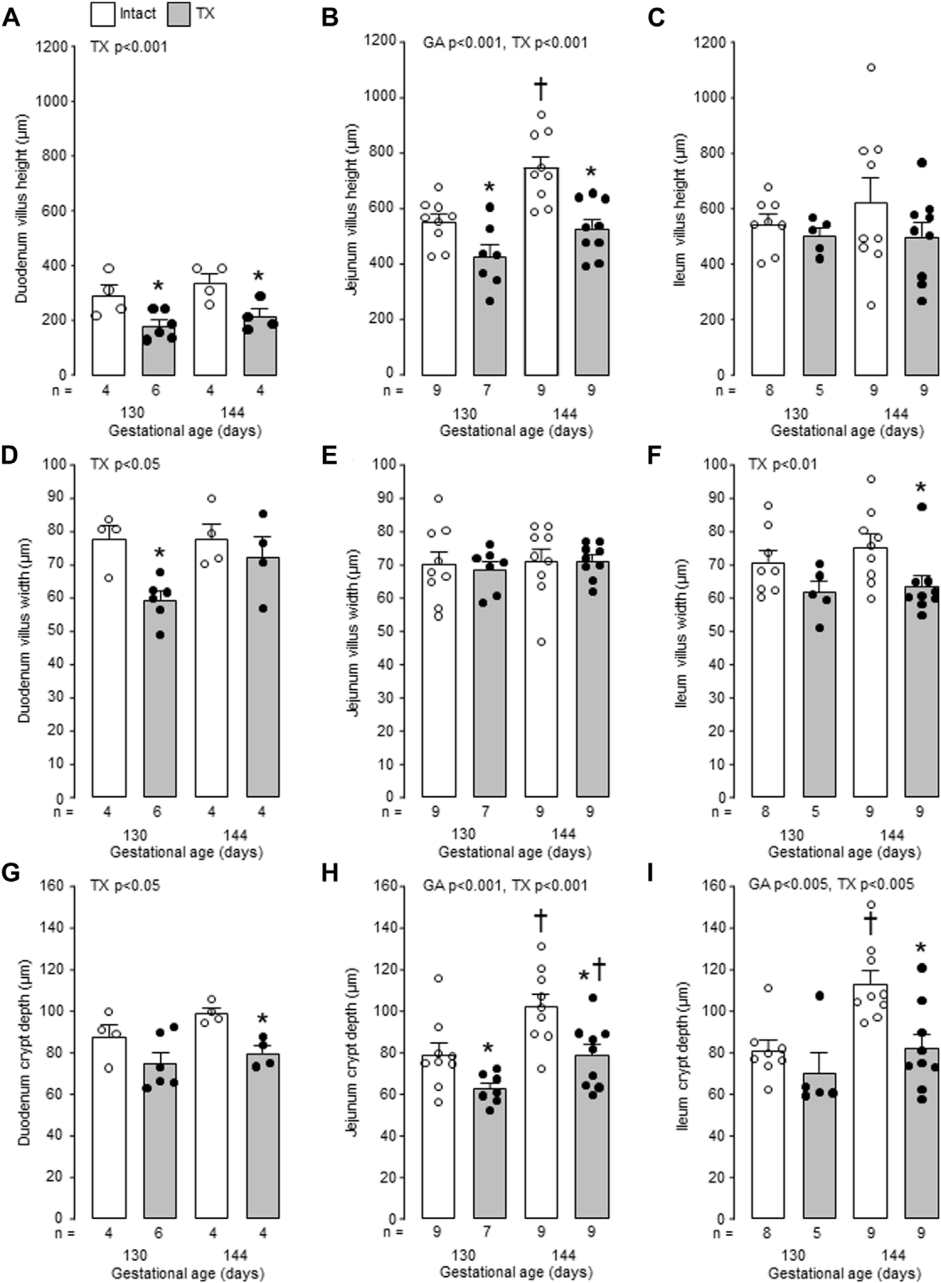
Mean (±SEM) and individual measurements of villus height **(A–C)**, villus width **(D–F)** and crypt depth **(G–I)** in the duodenum, jejunum and ileum of the intact and TX fetuses studied at 130 and 144 days of gestation. *, significant difference from intact fetuses at the same gestational age (*p* < 0.05, Tukey’s *post hoc* test following two-way ANOVA); ^†^, significant difference from fetuses of the same treatment at 130 days of gestation (*p* < 0.05, Tukey’s *post hoc* test following two-way ANOVA). Numbers of fetuses in each group are indicated at the base of the histogram. GA, gestational age; TX, thyroidectomy.

Mucosal villus height and crypt depth in all regions of the small intestines, and villus width in the duodenum and ileum, increased in the intact fetuses with gestational age (*p* < 0.05, Figure 6). Within the intestinal mucosa, aspects of normal villus-crypt development were impaired by hypothyroidism (Figure 7). Villus height was reduced in duodenum and jejunum, but not ileum, in the TX compared to intact fetuses at both 130 dGA and 144 dGA (*p* < 0.05, Figures 7A–C). In the TX fetuses, villus width was lower in the duodenum at 130 dGA and in the ileum at 144 dGA (*p* < 0.05) but remained unchanged in the jejunum (Figures 7D–F), and crypt depth was lower at 144 dGA in the duodenum and ileum and at both 130 dGA and 144 dGA in the jejunum (*p* < 0.05, Figures 7G–I).

### Development of the intestinal submucosa and muscularis propria

In the intact fetuses studied between 100 and 144 dGA, the thickness of the intestinal submucosa was affected by gestational age in the ileum alone (*p* < 0.01, Table 1). Submucosal thickness in the ileum was also reduced by hypothyroidism (*p* < 0.05), and was lower in the TX fetuses compared with intact fetuses at 144 dGA (*p* < 0.05 in *post hoc* test, Table 1). In the jejunum, an interaction was observed between gestational age and treatment in submucosal thickness (*p* < 0.05), which was increased at 144 dGA by hypothyroidism (*p* < 0.05 in *post hoc* test, Table 1). The thickness of the muscularis propria was not affected by gestational age or hypothyroidism in any region of the small intestines (Table 1).

### Intestinal enzyme activity

The activity of intestinal brush border enzymes in the intact fetuses depended on gestational age, region of the intestines and specific enzyme investigated. Between 100 and 144 dGA, gestational age influenced the activities of sucrase and lactase in the duodenum and jejunum, ApN in the jejunum and ileum, and maltase and ApA in the ileum (*p* < 0.05, Table 2).

**TABLE 2:** Mean (±SEM) activities of intestinal enzymes in the fetuses of each experimental group at tissue collection. One-way ANOVA assessed the effect of gestational age in the intact fetuses between 100 and 144 days of gestation: for each parameter measured, values with different superscript letters are significantly different from each other (*p* < 0.05, Tukey’s *post hoc* test). Two-way-ANOVA examined the effects of gestational age and TX in the intact and TX fetuses studied at 130 and 144 days of gestation: *, significant difference from intact fetuses at the same gestational age (*p* < 0.05, Tukey’s *post hoc* test); ^†^, significant difference from fetuses of the same treatment at 130 days of gestation (*p* < 0.05, Tukey’s *post hoc* test). GA, gestational age; TX, thyroidectomy; ApA, aminopeptidase A; ApN, aminopeptidase N; DPPIV, dipeptidyl peptidase IV n, number of animals; NS, not significant.

	Effect of gestational age in intact fetuses					Effect of gestational age and TX						
Gestational age (days)	100	115	130	144	GA	130		144		GA	TX	Interaction
Treatment	intact	intact	intact	intact		intact	TX	intact	TX			
**Duodenum**	n = 4	n = 4	n = 12	n = 12		n = 12	n = 13	n = 12	n = 14			
Sucrase (U/g tissue)	0.08 ± 0.01^A^	0.11 ± 0.01^AB^	0.15 ± 0.01^B^	0.13 ± 0.01^AB^	*p* < 0.005	0.15 ± 0.01	0.12 ± 0.01	0.13 ± 0.01	0.13 ± 0.01	NS	NS	NS
Maltase (U/g tissue)	0.39 ± 0.02	0.41 ± 0.01	0.53 ± 0.04	0.48 ± 0.04	NS	0.53 ± 0.04	0.48 ± 0.04	0.48 ± 0.04	0.44 ± 0.04	NS	NS	NS
Lactase (U/g tissue)	19.7 ± 2.0^A^	38.8 ± 4.8^AB^	59.8 ± 7.2^B^	42.7 ± 7.5^AB^	*p* < 0.05	59.8 ± 7.2	51.0 ± 5.9	42.7 ± 7.5	43.0 ± 6.1	NS	NS	NS
ApA (U/g tissue)	0.13 ± 0.02	0.15 ± 0.01	0.17 ± 0.01	0.15 ± 0.01	NS	0.17 ± 0.01	0.16 ± 0.01	0.15 ± 0.01	0.17 ± 0.01	NS	NS	NS
ApN (U/g tissue)	2.06 ± 0.23	1.54 ± 0.16	2.00 ± 0.23	2.74 ± 0.42	NS	2.00 ± 0.23	1.76 ± 0.09	2.74 ± 0.42	2.78 ± 0.28^†^	*p* < 0.005	NS	NS
DPPIV (U/g tissue)	1.76 ± 0.12	1.88 ± 0.27	1.44 ± 0.14	1.52 ± 0.14	NS	1.44 ± 0.14	1.59 ± 0.15	1.52 ± 0.14	1.87 ± 0.16	NS	NS	NS
**Jejunum**	n = 5	n = 5	n = 16	n = 12		n = 16	n = 14	n = 12	n = 14			
Sucrase (U/g tissue)	0.10 ± 0.03^AB^	0.09 ± 0.02^A^	0.21 ± 0.03^B^	0.18 ± 0.02^AB^	*p* < 0.05	0.21 ± 0.03	0.18 ± 0.02	0.18 ± 0.02	0.14 ± 0.02	NS	NS	NS
Maltase (U/g tissue)	0.82 ± 0.17	0.79 ± 0.17	0.90 ± 0.09	0.89 ± 0.10	NS	0.90 ± 0.09	1.00 ± 0.15	0.89 ± 0.10	0.79 ± 0.10	NS	NS	NS
Lactase (U/g tissue)	40.9 ± 17.9^A^	104.7 ± 10.7^B^	94.4 ± 12.8^AB^	61.6 ± 9.3^A^	*p* < 0.05	94.4 ± 12.8	71.1 ± 11.8	61.6 ± 9.3^†^	58.2 ± 6.9	*p* < 0.05	NS	NS
ApA (U/g tissue)	0.64 ± 0.09	0.78 ± 0.13	0.94 ± 0.09	0.63 ± 0.09	NS	0.94 ± 0.09	0.9 ± 0.12	0.63 ± 0.09	1.04 ± 0.15	NS	NS	NS
ApN (U/g tissue)	42.7 ± 7.7^AB^	31.4 ± 4.1^A^	63.6 ± 5.6^AB^	96.4 ± 16.2^B^	*p* < 0.005	63.6 ± 5.6	80.5 ± 9.4	96.4 ± 16.2^†^	98.6 ± 8.3	*p* < 0.05	NS	NS
DPPIV (U/g tissue)	4.2 ± 0.5	5.33 ± 1.09	6.57 ± 0.67	5.64 ± 0.47	NS	6.57 ± 0.67	6.94 ± 0.86	5.64 ± 0.47	6.21 ± 0.32	NS	NS	NS
**Ileum**	n = 5	n = 5	n = 18	n = 14		n = 18	n = 14	n = 14	n = 14			
Sucrase (U/g tissue)	0.08 ± 0.02	0.15 ± 0.05	0.11 ± 0.01	0.14 ± 0.01	NS	0.11 ± 0.01	0.12 ± 0.01	0.14 ± 0.01	0.11 ± 0.02	NS	NS	NS
Maltase (U/g tissue)	0.47 ± 0.21^A^	0.61 ± 0.24^AB^	0.87 ± 0.13^AB^	1.35 ± 0.17^B^	*p* < 0.05	0.87 ± 0.13	0.99 ± 0.11	1.35 ± 0.17^†^	0.80 ± 0.11*	NS	NS	*p* < 0.05
Lactase (U/g tissue)	13.7 ± 4.1	14.7 ± 2.6	17.3 ± 2.4	18.5 ± 2.1	NS	17.3 ± 2.4	19.0 ± 3.2	18.5 ± 2.1	18.5 ± 2.1	NS	NS	NS
ApA (U/g tissue)	3.96 ± 0.33^A^	3.59 ± 0.49^AB^	1.56 ± 0.23^BC^	1.35 ± 0.31^C^	*p* < 0.001	1.56 ± 0.2	1.75 ± 0.32	1.35 ± 0.31	1.89 ± 0.54	NS	NS	NS
ApN (U/g tissue)	289.3 ± 29.2^A^	169.1 ± 23.6^B^	158.2 ± 11.5^B^	141.3 ± 20.3^B^	*p* < 0.001	158.2 ± 11.5	256.5 ± 33.2*	141.3 ± 20.3	203.8 ± 24.9	NS	*p* < 0.001	NS
DPPIV (U/g tissue)	7.13 ± 1.43	7.05 ± 0.47	6.69 ± 0.44	8.38 ± 0.81	NS	6.69 ± 0.44	7.29 ± 0.71	8.38 ± 0.81	5.18 ± 0.68*^†^	NS	*p* < 0.05	*p* < 0.005

When data from the intact and TX fetuses at 130 dGA and 144 dGA were assessed, gestational age affected ApN activities in the duodenum and jejunum, and lactase in the jejunum (*p* < 0.05, Table 2). *Post-hoc* analysis showed that duodenal ApN increased in the intact (*p* = 0.06) and TX fetuses, and jejunal lactase decreased and ApN increased in the intact fetuses, near term (all *p* < 0.05, Table 2). Enzyme activities in the duodenum and jejunum, however, were unaffected by hypothyroidism (Table 2).

In the ileum, interactions between gestational age and treatment were observed for maltase and DPPIV enzyme activities (*p* < 0.05, Table 2). Between 130 dGA and 144 dGA, ileal maltase increased in the intact, but not TX fetuses, and DPPIV enzyme activity decreased in the TX fetuses (*p* < 0.05, Table 2). For both maltase and DPPIV in the ileum, enzyme activities were lower in the TX compared with intact fetuses at 144 dGA (*p* < 0.05, Table 2). Ileal ApN enzyme activity, however, was increased by hypothyroidism (*p* < 0.05): ApN activity was greater in TX than intact fetuses at 130 dGA (*p* < 0.05) with a similar tendency at 144 dGA (*p* = 0.06, Table 2).

## Discussion

Thyroid hormone deficiency in the ovine fetus impaired the structural and functional development of the gastrointestinal tract seen normally near term. Hypothyroidism caused growth retardation of the mucosa in the abomasum and small intestines, and reduced maltase and DPPIV enzyme activities in the ileum, in association with lower circulating concentrations of cortisol and gastrin. These effects on gastrointestinal development are likely to have consequences for digestive function in the neonatal period and later life.

The present findings are novel in a model relevant to the human fetus and are similar to those seen over the early postnatal period in more altricial rodent species. In rat pups thyroidectomised at 6 days after birth, intestinal villi and crypts showed little growth over the subsequent 2–3 weeks and the relative weight of the small intestines was reduced to 72% of that in the control animals (Yeh and Moog, 1977). Intestinal maltase and sucrase activities were also suppressed in the TX rat pups, and both the structural and digestive enzyme parameters were improved by treatment with glucocorticoids and T4 (Yeh and Moog, 1977). Over the same postnatal time-frame, the numbers of proliferating cells in the intestinal epithelium were reduced in mouse models of congenital hypothyroidism (Pax8^−/−^, Flamant et al., 2002), deletion of the thyroid hormone receptor-α (THR-α^−/−^, Plateroti et al., 2006) and pharmacological thyroid hormone deficiency (propylthiouracil and low iodine, Plateroti et al., 2001). In mouse pups, deletion of THR-α and pharmacological hypothyroidism were both associated with downregulation of key genes in the control of proliferation and differentiation pathways in the small intestines, such as Cyclin D1, *β*-Catenin, and Cdx1 and Cdx2 homeobox genes (Fraichard et al., 1997; Plateroti et al., 1999, 2001; 2006).

Before birth, hypothyroidism may influence the growth and development of the gastrointestinal tract by a variety of mechanisms, and the findings of the present study indicate important interactions between thyroid hormones, cortisol and gastrin in regulating these processes. Plasma cortisol concentration was reduced in the TX ovine fetus near term and this has been shown previously to be due to reductions in circulating adrenocorticotrophic hormone (ACTH), the size of the adrenal zona fasciculata and expression of steroidogenic enzymes (Camm et al., 2021). Cortisol has growth-promoting and maturational effects in the developing gastrointestinal tract and elevates circulating gastrin concentration in fetal pigs and sheep (Trahair et al., 1987a; Sangild T. et al., 1994, 1995b). Therefore, growth retardation and dysmaturation of the gastrointestinal tract may, in part, be attributed to the moderately lower circulating level of glucocorticoids in the TX fetus. In rats, hypothyroidism over the perinatal period also decreases the expression of components of the hypothalamic-pituitary-adrenal (HPA) axis and ACTH and glucocorticoid secretion (Dakine et al., 2000), although the relative contribution of suppressed glucocorticoid activity to the gastrointestinal abnormalities seen in models of fetal or neonatal hypothyroidism is unknown. Further investigations require the use of targeted thyroid hormone deficiency in the developing gastrointestinal tract without consequence for the HPA axis.

Gastrin-secreting G-cells are present primarily in the pyloric antrum and duodenum of sheep fetuses from mid-gestation, and antral and circulating gastrin concentrations increase with gestational age to peak at birth (Read et al., 1992; Franco et al., 1993). In the present study, the lack of rise in plasma gastrin near term induced by thyroid hormone deficiency suggests that T3 may regulate gestational and cortisol-induced increments in G-cell development and plasma gastrin concentration (Sangild T. et al., 1994). Fasting plasma gastrin, and responses to arginine injection, were lower in hypothyroid adult human patients and improved with thyroid hormone treatment, such that plasma gastrin correlated with T3 concentration (Seino et al., 1978; Sagara et al., 1983). Low plasma gastrin concentration in the TX ovine fetus may reflect the reduced size of the abomasal and duodenal mucosa and, in turn, may contribute to the impaired growth and development of the gastrointestinal tract. Removal of the abomasal antrum in fetal sheep at 90 dGA, which decreases plasma gastrin to less than half of the normal concentration, was associated with lower villi and crypt densities in the small intestine at 135 dGA (Avila et al., 1989). In this model of hypogastrinaemia, there were no changes in total gastrointestinal weight, thickness of the mucosa in the remaining abomasal fundus, or intestinal villus-crypt structure, although fetuses were not studied over the last 1–2 weeks of gestation when marked changes in gastrointestinal development take place (Avila et al., 1989; Buddington et al., 2012; Flores et al., 2018). The extent to which plasma gastrin plays a role in the maturation of the fetal gastrointestinal tract induced by glucocorticoids and thyroid hormones, or merely indicates G-cell number and secretory function, remains to be established.

Before birth, swallowing and passage of amniotic fluid, containing growth factors, hormones and nutrients, is critical for normal growth of the gastrointestinal tract, the activity of brush border enzymes and the development of nutrient uptake mechanisms (Kimble et al., 1999; Sangild et al., 2002; Cellini et al., 2006). Growth factor expression within the developing gastrointestinal tract may be impaired by hypothyroidism as observed previously in fetal ovine tissues such as the liver and skeletal muscle (Forhead et al., 2000, 2002). In fetal sheep, luminal infusion of IGF-I increased the weights of the whole stomach and intestines (Trahair et al., 1997), and in preterm piglets, subcutaneous IGF-I injections promoted intestinal growth, including villus height, and ApN and DPPIV activities, and reduced the appearance and severity of NEC lesions (Holgersen et al., 2020, 2022).

Although the size of the intestinal muscularis propria was unchanged in TX ovine fetuses, thyroid hormone deficiency may affect the innervation and contractility of smooth muscle in the gastrointestinal tract, and hence, luminal exposure to amniotic fluid along its length. Gastrointestinal hypomotility and prolonged transit time have been reported in newborn human infants with congenital hypothyroidism and in a case report of a child with a mutation in the THR-α gene (Smith et al., 1975; Bochukova et al., 2012). In colon segments taken from sheep fetuses treated with the synthetic glucocorticoid betamethasone, the muscle tension generated in response to a muscarinic agonist *in vitro* was greater when T4 was combined with the glucocorticoid (Ross et al., 1997). Similarly, fetal gut motility was accelerated by intra-amniotic injection of betamethasone and T4 in rhesus monkeys (Gilbert et al., 2001). Thyroid hormones are important for the development of both central and peripheral nervous systems (Williams, 2008; Prezioso et al., 2018), and have been shown to regulate proliferation and differentiation of progenitor cells of the enteric nervous system taken from neonatal mice and studied *in vitro* (Mohr et al., 2013). Furthermore, in zebrafish larvae, pharmacological hypothyroidism suppresses intestinal transit in association with a reduction in proliferating and total number of enteric neurones; these changes are restored to normal by T4 supplementation (Wang et al., 2020). Further experiments are needed to establish the extent to which the development of the enteric nervous system and control of gastrointestinal motility is influenced by hypothyroidism in the ovine fetus before birth with consequences for the maturation of the gastrointestinal tract.

In the present study, hypothyroidism *in utero* did not affect the normal ontogenic changes seen in enzyme activities in the duodenum or jejunum, some of which correlated with structural indices in these regions of the fetal small intestines. In the intact control fetuses, many of the enzymes were altered before the prepartum period and, therefore, appeared not related simply to growth of the intestinal mucosa or changes in circulating glucocorticoid or thyroid hormone concentrations. Ileal maltase and DPPIV activities, however, were suppressed in the TX compared to intact fetuses near term. For ileal maltase, the effects of hypothyroidism may have been mediated by relationships between enzyme activity and indices of villus-crypt architecture. In contrast, ileal ApN activity was increased by thyroid deficiency at both gestational ages studied which suggests that thyroid hormones may normally exert an inhibitory effect on ApN activity in the distal region of the small intestine. Regional and enzyme-specific responses to hypothyroidism may relate to differences in thyroid hormone signalling along the gastrointestinal tract which require further investigation. In rat pups studied at 3 weeks of postnatal age, pharmacological hypothyroidism suppressed intestinal maltase and sucrase activities and these were restored by T4 injections (Martin and Henning, 1982). Similarly, thyroidectomy further delayed the maturation of intestinal maltase, sucrase and lactase activities in adrenalectomized rat pups over the same postnatal period (Koldovský et al., 1975). Maternal T3 treatment in rats increased intestinal maltase and sucrase in the offspring, both before birth and during the suckling period (Jumawan et al., 1977; Koldovský et al., 1980; Vaucher et al., 1982).

The present study indicates the complexity of endocrine interactions in the development of the fetal gastrointestinal tract near term. Here, hypothyroidism *in utero* primarily impaired growth of the ovine gastrointestinal tract, whereas previous studies of cortisol treatment in fetal pigs have shown more marked effects on functional maturation of brush border enzymes and other digestive secretions (Sangild et al., 1994; 1995a). This suggests that the prepartum rises in both T3 and cortisol are important for the coordinated structural and functional maturation of the digestive system in the preparation for birth, as seen in other fetal tissues (Forhead and Fowden, 2014; Fowden et al., 2016). However, there may also be species differences in the nature and timeframe of development in the proximal and distal regions of the gastrointestinal tract in animals that will become either fore-gut (sheep) or hind-gut (pig) fermenters. Indeed, it will be intriguing to examine how hormones before birth prepare the environment for, and influence the profiles of, the microbiota that colonise the gut after the onset of enteral feeding.

Marked structural and functional changes in the gastrointestinal tract occurred in the intact control fetuses over the last quarter of gestation, similar to those reported previously in sheep and pigs (Trahair and Sangild, 1997; Buddington et al., 2012). These findings highlight the importance of this period for the onset of normal digestive function at birth and indicate the likely consequences for offspring born preterm. Growth retardation of the mucosa and villus-crypt structure, and altered digestive enzyme activities, in the TX ovine fetus will also influence the surface area and the secretory and absorptive capacity of the gastrointestinal tract and, in turn, the systemic acquisition of nutrients. However, the effects of prematurity and/or hypothyroidism on the expression of nutrient and other transporters, and on the secretory and uptake mechanisms in gastric cells and enterocytes, remain to be established in the ovine fetus. The findings of this study have implications for the understanding of gastrointestinal pathologies in infants with congenital hypothyroidism and those born preterm with low circulating thyroid hormone concentrations (Fisher, 2007). Use of antenatal synthetic glucocorticoids in preterm delivery promotes an earlier onset of enteral nutrition and reduces the risk of NEC in the premature human infant (Halac et al., 1990; Jing et al., 2021). In sheep fetuses, maternal dexamethasone treatment is associated with increased circulating T3 concentration which may, in part, contribute to the maturational effects of the synthetic glucocorticoids (Forhead et al., 2007). Thyroid hormones, therefore, have an important role in the coordinated control of growth and maturation of the fetal gastrointestinal tract near term and in the clinical management of prematurity.

## Data Availability

The raw data supporting the conclusion of this article will be made available by the authors, without undue reservation.
